# Supraclavicular transposition of aberrant left vertebral artery for hybrid treatment of aortic arch aneurysm: a case report

**DOI:** 10.1186/s13019-017-0574-8

**Published:** 2017-01-31

**Authors:** Kyo Seon Lee, Gwan Sic Kim, Yochun Jung, In Seok Jeong, Kook Joo Na, Bong Suk Oh, Byung Hee Ahn, Sang Gi Oh

**Affiliations:** Department of Thoracic and Cardiovascular Surgery, Chonnam National University Hospital, Chonnam National University School of Medicine, 42, Jebong-ro, Dong-gu, Gwangju, 15772 South Korea

**Keywords:** Aortic arch, Aortic operation, Stents, Case report

## Abstract

**Background:**

Vertebral artery variations are common in thoracic aortic patients. If patients have the aberrant left vertebral artery, the more difficult to determine the treatment modality.

**Case presentation:**

We report the case of a 63-year-old man with an aberrant left vertebral artery originating from an aneurysmal aortic arch. The patient underwent a successful hybrid thoracic endovascular aortic repair after aortic arch debranching and transposition of the aberrant left vertebral artery to the left common carotid artery through a supraclavicular incision without sternotomy.

**Conclusions:**

The aberrant left vertebral artery originating from the aortic arch can be safely transposed to the left common carotid artery through a supraclavicular approach.

## Background

Although stent-grafts have evolved in recent years, the management of aortic arch disease remains to be difficult due to the arch vessels. In about 40% of patients undergoing thoracic endovascular aortic repair (TEVAR), the left subclavian artery (LSA) must be intentionally covered in order to obtain an adequate proximal landing zone and achieve stable sealing [1]. However, if patients have the aberrant left vertebral artery, the more difficult to determine the treatment modality. We describe a case treated with a novel hybrid TEVAR technique without sternotomy.

## Case presentation

A 65-year-old man presenting with chest pain and back pain was transferred to our hospital for further evaluation of aortic arch aneurysm on chest radiograph. He had been taking anti-hypertensive for 2 years. On admission, his heart rate was 76 beats per minute in sinus rhythm and blood pressure was 110/70 mmHg.

**Fig. 1:**
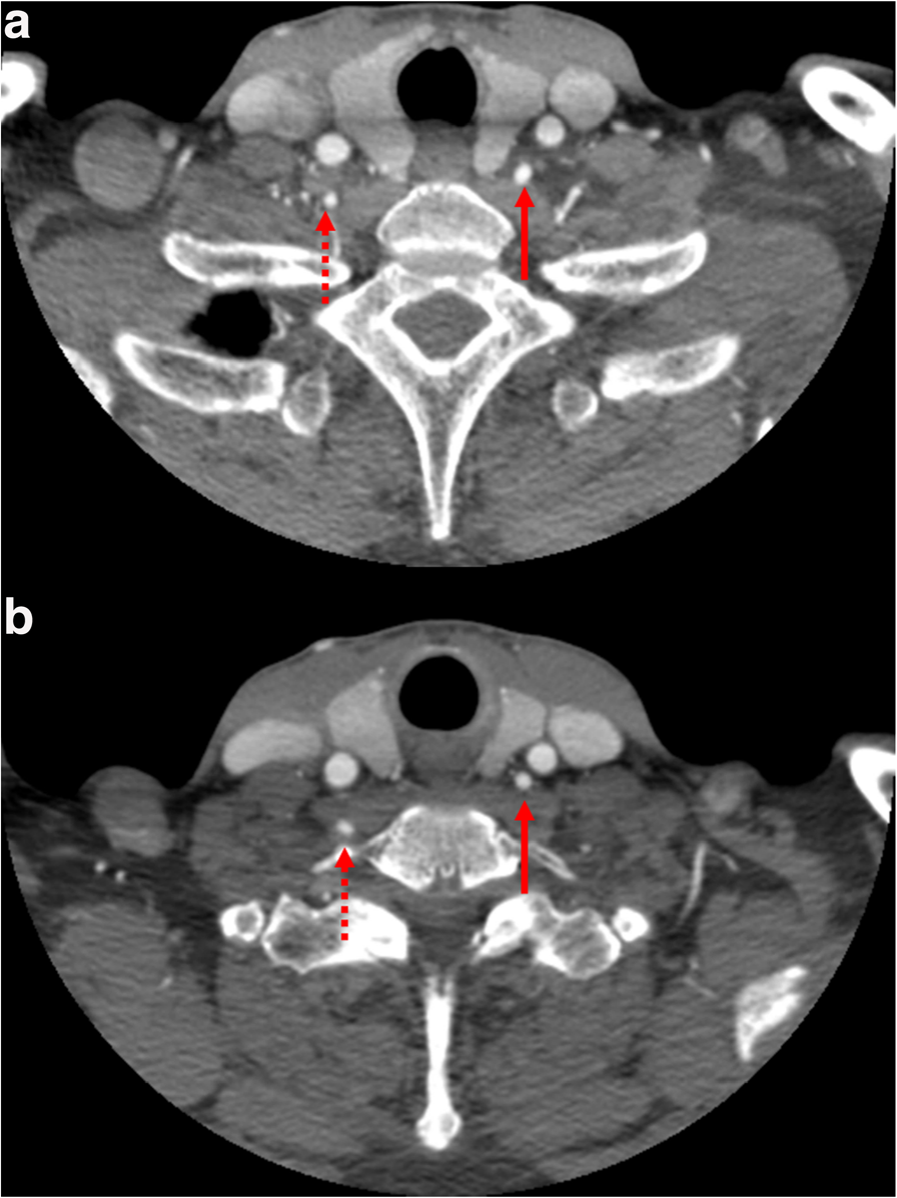
**a**, **b** Preoperative neck computed tomographic (CT) angiography. Solid arrow: the aberrant left vertebral artery; dotted arrow: the right vertebral artery

Chest computed tomography (CT) was performed and revealed a thrombosed aneurysm of the aortic arch. Maximal diameter was 62 mm. The aneurysm extended from immediately distal to the left common carotid artery (LCA) up to the distal aortic arch, and the aberrant left vertebral artery originated from just proximal aortic arch segment with aneurysmal change. Neck and brain CT was performed to evaluate the neck vessels and the intracranial arteries. There was no occlusive lesion of the neck vessels, including both vertebral arteries, and the continuity of the circle of Willis was intact. However, there was hypoplasia on the left vertebral artery at T1 level (Fig. 1).

**Fig. 2:**
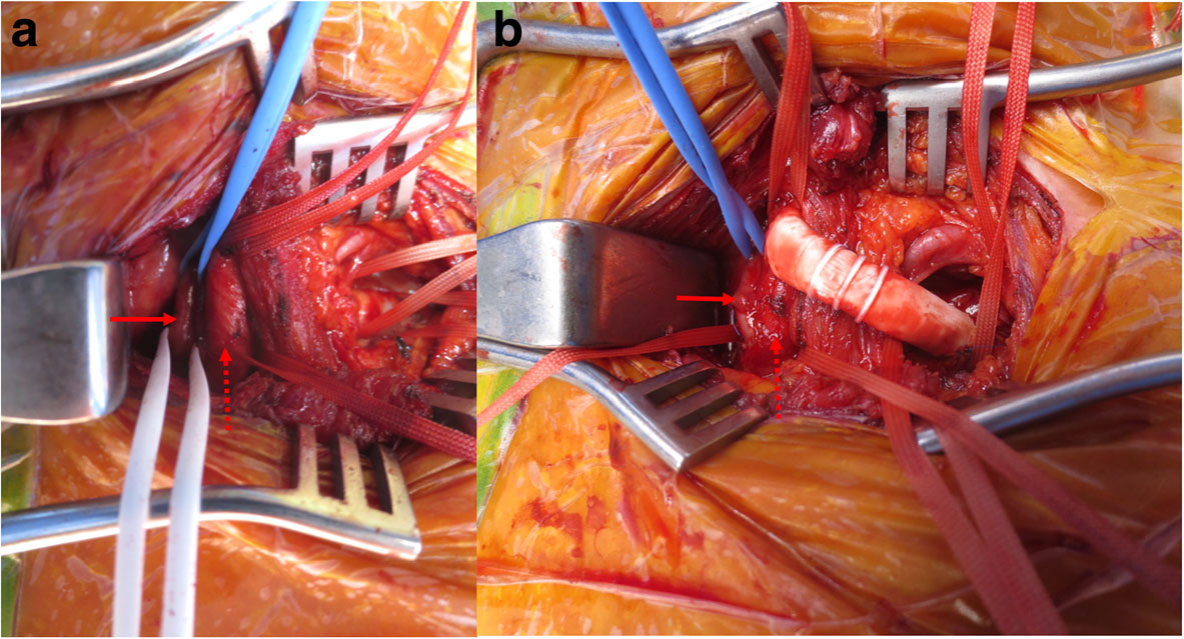
Intraoperative photographs of left supraclavicular incision. **a** Isolation of the left common carotid artery (dotted arrow) and aberrant left vertebral artery (solid arrow). **b** Final anastomosis between the left subclavian artery and the left common carotid artery (dotted arrow) using vascular graft, and transposed the aberrant left vertebral artery (solid arrow) to the left common carotid artery

The patient underwent arch vessel debranching surgery through a supraclavicular approach. The left supraclavicular incision was performed and the LSA and the LCA were isolated. A 7 mm ringed vascular graft (GORE-TEX® vascular graft FEP removable ring 7 mm-70 cm, W.L. Gore & Associates Inc., USA) was used to anastomose the LSA to the LCA. The aberrant left vertebral artery was transposed to the LCA (Fig. 2). The proximal LSA was ligated to prevent endoleak. Next, endovascular stent-graft placement was performed after 2 days later. Using a right femoral artery cut-down and percutaneous left femoral artery access, the stent-graft (VALIANT THORACIC® stent graft 40–150, Medtronic., USA) was implanted, extending from zone 2 to the upper descending thoracic aorta, 5 cm distal to the aneurysm.

**Fig. 3:**
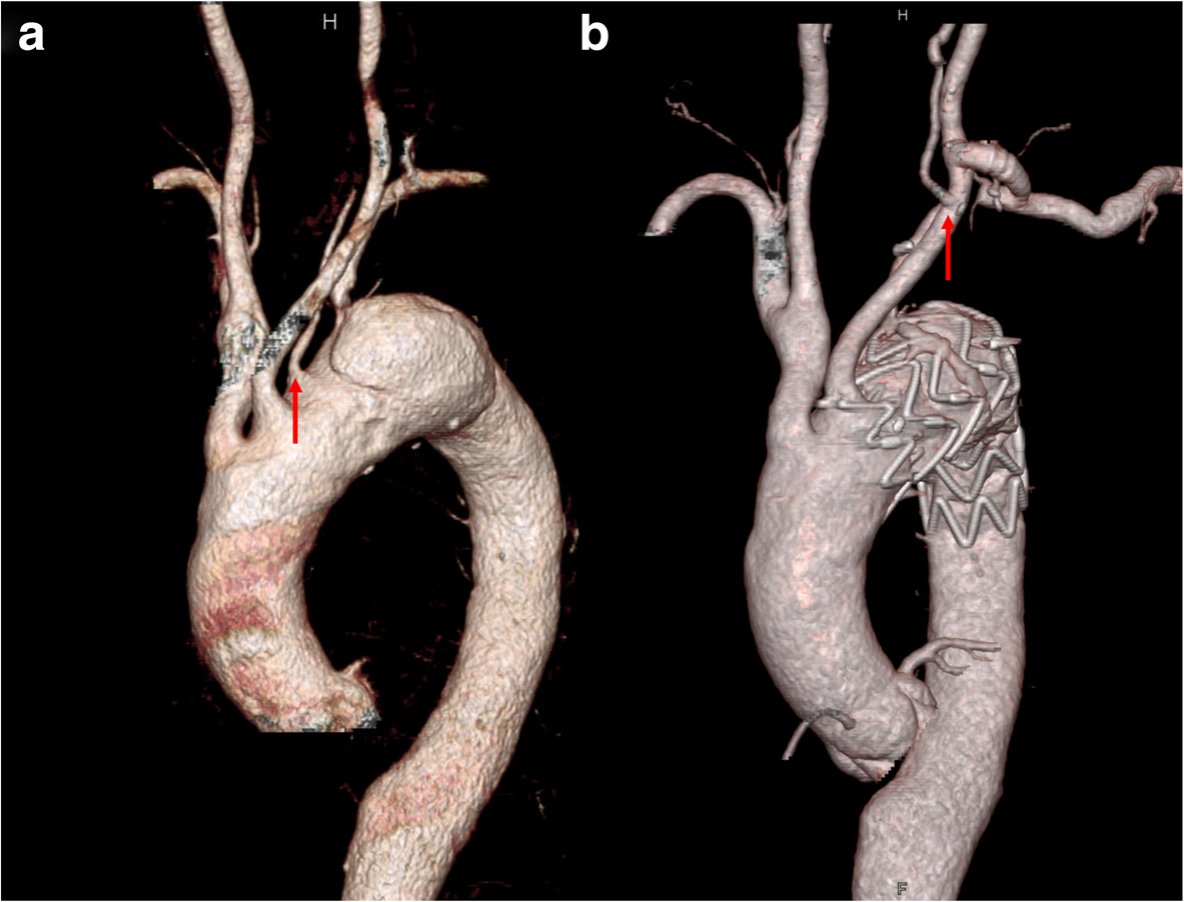
Chest computed tomographic (CT) angiography. **a** Preoperative CT angiography, the aberrant left vertebral artery (solid arrow) from aortic arch **b** Postoperative CT angiography, the transposed left vertebral artery (soild arrow) to the left common carotid artery

The patient was discharged without complications. Follow-up CT revealed patent arch vessels, including the transposed left vertebral artery (Fig. 3).

There is increasing evidence pointing to the need for the LSA revascularization in order to prevent cerebral, spinal cord, and left arm ischemia; on the other hand, opposing opinion argues that the evidence is low-quality and the actual incidence of ischemia is rare [2, 3]. Nevertheless, in selected patients, specifically those who have a patent left internal thoracic artery to coronary artery bypass graft, a functioning arteriovenous shunt in the left arm, or presence of vertebral artery variations like hypoplasia or discontinuity of the vertebrobasilar collaterals, the LSA revascularization is strongly recommended [1]. The vertebral arteries converge into the basilar artery and it supplies blood to the brainstem, cerebellum and occipital lobes. In addition, the segmental arteries that supply the spinal cord arise from the vertebral arteries. Therefore, the LSA revascularization is important in order to prevent cerebrospinal ischemia in patients with normal left vertebral arteries originating from the LSA. At present, there is no clear recommendation on whether an aberrant left vertebral artery should be revascularized or not. While the LSA should ideally be revascularized to prevent arm ischemia, revascularization of an aberrant left vertebral artery may be omitted if the right vertebral artery is normal [3]. Vertebral artery variations are common in thoracic aortic patients, with the right vertebral artery being particularly more variable than the left, having a prevalence of up to 30% [4]. Although the patient’s right vertebral artery was patent in the brain and neck CT scan, we decided to transpose the left vertebral artery from the arch to the LCA because hypoplasia of the right vertebral artery was suspected. At the same time, he suffers from hypertension, which can increase the risk of stroke in the future. Furthermore, the neck CT scan revealed that the left vertebral artery did not enter the transverse foramen at the level of C6, but instead at a higher level at C4, coursing posterior to the LCA. Melia et al. demonstrated this course of the extracranial vertebral artery in patients with abnormal left vertebral arteries [5].

The left supraclavicular incision is the most commonly used approach when performing an LSA bypass. This approach has long been used for the treatment of arch vessel diseases by neurosurgeons [6]. The LSA is easily manageable by this approach and the aberrant left vertebral artery originating from the aortic arch is readily accessible because of its parallel course to the LCA (Fig. 1). In a recent study, although the LSA transposition to LCA showed longer patency than the LSA bypass using artificial graft, there was no difference between two methods [7, 8]. Furthermore, to obtain an adequate length for LCA transposition, the LCA should be transected to as proximal a location as possible. Since this can affect the weakened disease-aortic arch, we prefer the LCA bypass.

More complex arch reconstruction requires a sternotomy, especially zone 0 procedures. This approach is familiar to thoracic surgeons, but is more invasive than the supraclavicular approach. Few reports on aberrant left vertebral artery transpositions exist, and to our knowledge, this is the only report of hybrid TEVAR with transposition of an aberrant left vertebral artery through a supraclavicular approach [9, 10].

## Conclusions

An aberrant left vertebral artery originating from the aortic arch can be safely transposed to the LCA through a supraclavicular approach. It is expected that this technique would expand the boundaries of TEVAR.

## Abbreviations

CT: Computed tomography
LCA: Left common carotid artery
LSA: Left subclavian artery
TEVAR: Thoracic endovascular aortic repair
